# Noninvasive imaging of oral neoplasia with a high-resolution fiber-optic microendoscope

**DOI:** 10.1002/hed.21735

**Published:** 2011

**Authors:** Timothy J Muldoon, Darren Roblyer, Michelle D Williams, Vanda MT Stepanek, Rebecca Richards–Kortum, Ann M Gillenwater

**Affiliations:** 1 Rice University Department of Bioengineering6100 Main Street, Houston, Texas 77005 E-mail: rkortum@rice.edu; 2 Department of Pathology, The University of Texas MD Anderson Cancer Center1515 Holcombe Boulevard, Houston, Texas 77030; 3 Department of Head and Neck Surgery, The University of Texas MD Anderson Cancer Center1515 Holcombe Boulevard, Houston, Texas 77030

**Keywords:** microendoscopy, optical imaging, dysplasia, computer aided diagnostics, fluorescence imaging

## Abstract

**Background:**

The purpose of this study was to evaluate the ability of high-resolution microendoscopy to image and quantify changes in cellular and architectural features seen in early oral neoplasia in vivo.

**Methods:**

A high-resolution microendoscope (HRME) was used to image intact, resected oral squamous carcinoma specimens. HRME images were reviewed and classified as non-neoplastic or neoplastic by expert clinicians. An algorithm based on quantitative morphologic features was also used to classify each image. Results were compared to the histopathologic diagnosis.

**Results:**

HRME images were obtained from 141 sites in resected specimens from 13 patients. Subjective image interpretation yielded sensitivity and specificity of 85% to 90% and 80% to 85%, respectively, whereas the objective classification algorithm achieved sensitivity and specificity of 81% and 77%, respectively.

**Conclusion:**

High-resolution microendoscopy of intact oral mucosa can provide images with sufficient detail to classify oral lesions by both subjective image interpretation and objective image analysis. © 2011 Wiley Periodicals, Inc. Head Neck, 2011

In the United States, the overall 5-year survival rate for patients with squamous carcinoma of the oral cavity is only 54%, 1 of the lowest rates of all major cancers; in developing countries, survival rates drop below 30%.^1^,^2^ Patients with early lesions have better chances for cure and less treatment-associated morbidity, yet despite the easy accessibility of the mouth, most patients present with advanced tumors, when treatment is more difficult, more expensive, and less successful compared to earlier interventions.^3^ Improved screening and early diagnosis of oral cancer could benefit the global population substantially.

Optical imaging technologies have the potential to improve early detection of oral neoplasia by noninvasively assessing the morphologic and biochemical changes associated with precancer and cancer in real time at the point-of-care.^4^–^6^ Driven by advances in consumer electronics, high quality optical images can now be obtained with low-cost devices; tandem advances in digital signal processing provide the ability to automate image analysis. These advances have the potential to improve screening in settings ranging from tertiary care centers to regions with limited personnel and infrastructure.

Enhanced imaging of broad areas of oral mucosa should enable clinicians to determine optimal sites for pinpoint probe interrogation and/or biopsy, reducing the chance of sampling error. Normally, clinicians observe reflected white light because this is the dominant light–tissue interaction and the intensity of reflected light far exceeds the intensity of induced fluorescence. However, it is also possible to directly observe tissue autofluorescence. Several groups have demonstrated that imaging systems that record the spatial distribution of fluorescence intensity at specific excitation/emission wavelength combinations can be used to survey large areas of oral cavity mucosa for neoplastic changes. A commercially available device, the VELscope (LED Dental, Inc., Burnaby, BC, Canada), uses a metal–halide lamp with emission peaks at 405 and 436 nm to excite autofluorescence; images indicate a characteristic loss of fluorescence associated with neoplasia.^7^,^8^ Results from 50 biopsies taken from areas with loss of fluorescence in 44 patients showed a sensitivity of 98% and specificity of 100% for discriminating normal tissue from severe dysplasia, carcinoma in situ, or invasive carcinoma, using histology as the gold standard. An important finding was the ability of fluorescence visualization to aid clinicians to see early neoplastic lesions that were initially missed during traditional white light examination.^9^ Additional results indicated that this device enhanced the ability of surgeons to visualize the peripheral extension of histologic and molecular abnormalities around neoplastic lesions to facilitate more accurate determination of resection margins.^10^

Although wide-field autofluorescence visualization can aid in the detection of early neoplastic changes, there is increasing concern that benign changes, such as inflammation, may also exhibit loss of fluorescence and may reduce specificity, especially in low-prevalence populations.^11^ High-resolution optical imaging can be used to directly visualize changes in epithelial morphology in suspicious regions of tissue, and can be used to complement such wide-field systems by distinguishing neoplastic from benign processes in regions with abnormal autofluorescence.^12^,^13^

A number of studies have explored the use of high-resolution optical imaging for improved detection of oral neoplasia.^14^,^15^ Reflectance confocal microscopy has been used to image changes in cell and nuclear morphology, nuclear-to-cytoplasmic ratio, and epithelial architecture associated with early oral neoplasia.^16^ A fiber-optic reflectance confocal microscope, comprising a single optical fiber and a resonating tuning fork at the distal tip, has been integrated into an endomicroscope platform to enable high-resolution fluorescence imaging of suspicious lesions in the oral cavity.^17^ Fiber-optic systems have also been developed to perform in vivo confocal fluorescence microscopy imaging. One such device, the Cellvizio (Mauna Kea Technologies, Paris, France) uses a galvanometric scanner to raster scan laser light across the proximal tip of a coherent fiber bundle to enable confocal fluorescence imaging of tissue at the distal tip of the device. This technique has been used successfully to image gastrointestinal and respiratory epithelium and other tissues in vivo.^18^,^19^ Although these fiber-optic confocal microscopes can obtain high-resolution images of tissue in real time, they are technically complex and expensive.

An alternative to confocal microscopy is high-resolution microendoscopy.^20^,^21^ The high-resolution microendoscope (HRME) uses a coherent fiber bundle to obtain high-resolution fluorescence images of the tissue in contact with the distal tip of the device without the need for complex mechanical scanning systems and associated control electronics.^22^,^23^ The system uses a low-cost light-emitting diode to provide illumination and a consumer-grade charge coupled device camera to capture high-resolution digital images on a laptop computer.

A topically applied fluorescent dye, proflavine (Sigma–Aldrich, St. Louis, MO), is used to preferentially stain cell nuclei. Proflavine is an acridine-derived dye which binds to DNA in a reversible and non-covalent manner.^24^,^25^ Proflavine has been safely used for years as 1 of the main components of triple dye, applied to the umbilicus of newborns to prevent infection.^26^ In addition, a number of in vivo imaging studies have been performed using topically delivered proflavine as a contrast agent.^27^,^28^ Proflavine is the principal component of acriflavine and has been used for fluorescence imaging in the European, Asian, and Australian gastrointestinal literature without any adverse effects noted.^27^ Moreover, proflavine has been used clinically as an antibacterial agent for decades. In neonatal care, triple dye, a combination of brilliant green, proflavine hemisulfate, and gentian violet is routinely used as a topical antibacterial agent on the umbilical stump of newborn babies,^26^ with a recent review of the practice categorizing toxicity as rare.^29^ The concentrations of proflavine solution required for successful imaging (0.01% to 0.05%)^27^ are substantially lower than that of the proflavine component in commercial triple dye, 0.11% (w/v; Kerr Triple Dye, VistaPharm). The quantity of solution required for diagnostic imaging is approximately the same as that used in neonatal care (0.65 mL per single-use swab). The additional exposure to light, which will occur during imaging, can also be compared to that received by newborn babies undergoing phototherapy for jaundice. The high-resolution fiber-optic microendoscope proposed for use here delivers 0.5 mW of 455 nm light to the tissue through a 0.8 mm diameter fiber-optic bundle, corresponding to an irradiance level of 100 mW/cm^2^. The American Academy of Pediatrics defines intensive phototherapy as a spectral irradiance of at least 30 μW/cm^2^ per nanometer over the 430 to 490 nm spectral band, equivalent to a total irradiance of 1.8 mW/cm^2^.^30^ Although the irradiance level is over 50 times higher with the fiber microendoscope system, a typical imaging session of 30 minutes (including imaging for routine care) is approximately 50 times shorter than a typical 24-hour (1440 minutes) phototherapy incubation, leading to an equivalent light dose in each scenario. Proflavine has also been safely used in previous clinical studies evaluating its effect as a photosensitizing agent for the treatment of genital herpes simplex virus.^31^ The ability of proflavine to stain epithelial cell nuclei is useful for precancer and cancer imaging applications because it allows for visualization of features such as nuclear-to-cytoplasmic ratio. Because digital images are acquired, quantitative analysis of this and other image metrics can be easily performed to aid in image analysis.^32^

Such high-resolution imaging techniques can complement wide-field autofluorescence imaging systems discussed earlier. Wide-field imaging devices, such as the VELScope, have the ability to survey large mucosal surface areas to detect regions with loss of autofluorescence, which are suspicious for dysplasia. The HRME device can complement these types of wide-field imaging systems by providing high-resolution image data at specific lesions first identified by loss of fluorescence. Other studies show that the type of data acquired by the HRME—information about the spatial distribution of nuclei in the epithelium—is less affected by removal from the body than is autofluorescence.^33^

The purpose of this study was to evaluate the ability of high-resolution microendoscopy to identify oral neoplasia. HRME images from each site were analyzed qualitatively by visual inspection and quantitatively using digital image analysis algorithms to determine whether the imaged site contained neoplastic tissue. Results of image analysis were compared to the gold standard of histopathology to assess the ability of HRME to correctly identify the presence of neoplasia.

## MATERIALS AND METHODS

### Patients and Data Collection

Patients over 18 years of age scheduled for surgery to remove a previously diagnosed oral squamous carcinoma or dysplasia were eligible to participate in the study. Informed consent was obtained from all study participants, and the study was reviewed and approved by the Institutional Review Boards at the University of Texas MD Anderson Cancer Center and Rice University. Patients underwent standard-of-care surgery to remove neoplastic oral lesions and associated margins. After surgical resection, the resected specimens were imaged.

**FIGURE 1:**
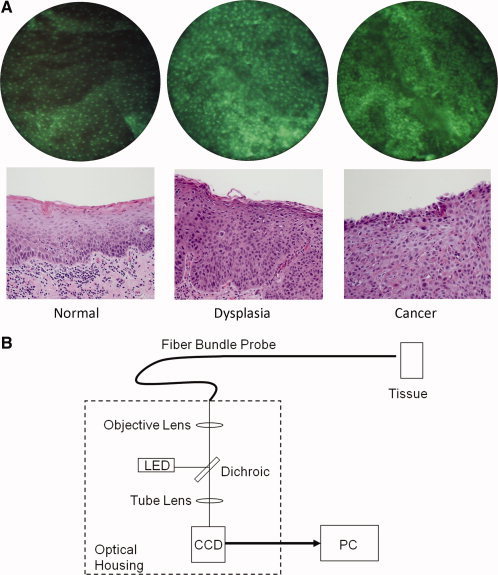
**(A)** Illustration of high-resolution microendoscope (HRME) images (top row) compared to hematoxylin-eosin images (bottom row). From left to right, diagnostic categories are: normal squamous mucosa, moderate to severe dysplasia, and invasive squamous carcinoma. All images are sized to the same scale; scale bar represents 100 μm. **(B)** Schematic diagram of HRME system.

A solution of proflavine (Sigma–Aldrich) dissolved in water at a concentration of 0.01% (w/v) was prepared before performing imaging. The contrast agent solution was directly applied to the epithelial surface of the resected tissue with a cotton-tipped swab, and imaging with the HRME device was performed immediately by gently placing the distal tip of the fiber-optic probe in contact with the epithelium. Because the intensity of proflavine fluorescence is approximately 10-fold higher than that of tissue autofluorescence, such autofluorescence does not interfere with imaging in tissue. The application of proflavine does not discolor the tissue surface, and is not detectable in tissue slides prepared using standard histopathology processing and hematoxylin-eosin staining (Figure 1A).

After imaging, the tissue was processed for standard histopathology, and slides were later reviewed by a board-certified pathologist (M.D.W.). Resected specimens typically ranged in size from 1 to 5 cm. After HRME images were obtained from each site, the mucosal surface of the entire specimen was photographed using a digital camera to document the location of the fiber probe at each imaged site. After imaging, specimens were returned to the pathology laboratory for histologic analysis. Different colors of ink were applied to each resected side of the tissue to facilitate registration of imaged sites and histopathology. Each specimen was then sliced transversely in a bread-loaf pattern. Each 1- to 5-mm thick transverse slice was then placed in a separately labeled slide cassette for processing. The photographs obtained during HRME imaging were used to identify the cassette corresponding to each imaged site. Hematoxylin-eosin slides were prepared from each cassette, and the precise location of each of the imaged sites was identified by noting the distance from each inked edge of the specimen. At each site imaged, the pathologic diagnosis was determined using 5 diagnostic categories (normal, mild dysplasia, moderate dysplasia, severe dysplasia, and cancer).

### High-Resolution Microendoscope System

The HRME device has been previously described in detail.^22^ Briefly, images are acquired with this device by placing the tip of the fiber bundle into direct contact with the epithelial surface of the tissue. Excitation light from a blue light-emitting diode with a center wavelength of 455 nm is delivered through the fiber bundle (Figure 1B). The fluorescence emission from the topically applied proflavine is collected through the fiber bundle, focused onto a charge coupled device camera, and a digital image is stored for future processing and analysis. The HRME system has a circular field of view with a diameter of 750 microns; the lateral spatial resolution of the system is approximately 4 microns.

### High-Resolution Microendoscope Image Analysis

HRME images were reviewed to determine whether the endoscope tip was in contact with the tissue surface or whether the probe tip moved during image acquisition. Images were assessed visually for quality control by a single observer who was blinded to the clinical impression and the histologic diagnosis. Images were discarded if they seemed out of focus, if there was evidence of motion artifact, or if it seemed that the fiber-optic probe was not in contact with the specimen when the image was acquired.

HRME images passing quality control were reviewed by a group of 3 observers with advanced knowledge of head and neck pathology from MD Anderson Cancer Center. Reviewers were first shown a small number of HRME images of the oral cavity acquired previously from sites with known histopathology; this training set consisted of images from 5 pathologically normal and 5 pathologically neoplastic measurement sites. Reviewers were subsequently asked to rate HRME images from all study sites; for each measurement, the observer was asked to rate the image on a 5-point scale, with 1 being “definitely non-neoplastic,” 2 being “probably non-neoplastic,” 3 being “unknown,” 4 being “probably neoplastic,” and 5 being “definitely neoplastic.”

To evaluate the accuracy of qualitative image interpretation, a 5-point receiver operator characteristic (ROC) curve was calculated, comparing the results of HRME image analysis to that of the gold standard of histopathology. A similar ROC analysis was conducted by Perednia et al^34^ to evaluate a dermatologists' subjective interpretations of digital images of skin lesions. To construct the ROC curve, the threshold for qualitative image analysis to be considered abnormal was varied from 1 to 5. The subjective rating at each measurement site was compared to this threshold, if the subjective rating was greater than or equal to the threshold, the image interpretation was considered “neoplastic.” Otherwise, the image interpretation for the measurement was considered “non-neoplastic.” This result was compared to the histopathology-confirmed diagnosis, sensitivity and specificity were calculated, and a 5-point ROC curve was constructed for each observer. Sites with a pathologic diagnosis of normal or mild dysplasia were considered to be non-neoplastic, whereas sites with a pathologic diagnosis of moderate dysplasia, severe dysplasia, or cancer were considered to be neoplastic.

We also explored the diagnostic ability of quantitative image analysis. All subsequent statistical analysis was performed using MATLAB (MathWorks, Natick, MA). A rectangular region of interest (ROI) was selected from the image of each independent site; ROIs were chosen from areas of the image illustrating cellular detail when present. The ROIs from each image were then used in the after quantitative analysis. A first-order statistical feature, entropy, was calculated directly from the histogram of the grayscale pixel values.^35^ Briefly, entropy is a scalar value that is a statistical measure of randomness in the pixel values of an image; its value can be used to characterize the texture of an image. It is well known that the texture of chromatin is altered with the presence of neoplasia.^36^ Moreover, the regular spacing of nuclei is altered with progression from dysplasia to carcinoma.^37^ The nuclear-to-cytoplasmic ratio, a physical measurement used by clinical pathologists, was estimated by manually thresholding each ROI until only nuclear features were visible. A binary image was then constructed, and pixels corresponding to nuclei were counted and divided by the total number of pixels in the ROI, yielding the nuclear-to-cytoplasmic ratio.^38^

The nuclear-to-cytoplasmic ratio was also calculated from selected ROIs taken directly from the histopathology slides for direct comparison to the HRME data. We identified a representative group of sites corresponding to each of the 5 histologic diagnostic categories. An ROI was selected which was approximately 100 microns wide by 20 microns tall, matching the measured depth of focus of the HRME system. This is necessary because the HRME images are en face with respect to the tissue, whereas the histopathology images are transversely oriented. To calculate the nuclear-to-cytoplasmic ratio at each ROI in the hematoxylin-eosin image, the digital images were segmented by hand and converted to a binary image, with white nuclei and black cytoplasm. The nuclear-to-cytoplasmic ratio was then calculated by dividing the number of pixels in the nuclear areas by the total area of the image.

A diagnostic algorithm was used to classify each ROI as non-neoplastic or neoplastic based on these image features. Histopathology was used as the gold standard; sites with a pathologic diagnosis of normal or mild dysplasia were considered to be non-neoplastic, whereas sites with a pathologic diagnosis of moderate dysplasia, severe dysplasia, or cancer were considered to be neoplastic. The classifier was based on 2-class linear discriminant analysis. The algorithm was developed using 5-fold cross-validation; initially, each measurement site was randomly assigned to 1 of 5 groups. Four-fifths of the data were then used to train the linear classification algorithm and the remaining one-fifth of the data were used to test the algorithm. This cycle was repeated 4 additional times so that the algorithm was tested using data from each site. Performance was monitored by calculating the area under the curve of the classifier.

## RESULTS

### Patients and Sites

Thirteen subjects were enrolled in the study. Images were obtained from 150 unique sites; images from 141 of these sites passed the quality control review and were used for further analysis. Of these 141 sites, 40 were histologically confirmed to be normal squamous tissue, 11 contained mild dysplasia, 11 contained moderate dysplasia, 12 contained severe dysplasia, and 67 contained invasive squamous carcinoma. Figure 1 shows representative HRME images (top row) of samples diagnosed as normal squamous epithelium, moderate dysplasia, and invasive cancer, respectively. Histopathology images can be seen below. All images are at the same magnification for comparison. The HRME image of normal squamous epithelium shows small, bright nuclei with dark cytoplasm and relatively large and evenly spaced internuclear separation. The image of dysplastic epithelium shows a marked increase in nuclear-to-cytoplasmic ratio and crowding of nuclei, and the image from invasive carcinoma shows high nuclear-to-cytoplasmic ratio and a distinct loss of cellular organization with haphazard nuclei with unevenly spaced large nuclei.

### Subjective Image Interpretation

**FIGURE 2:**
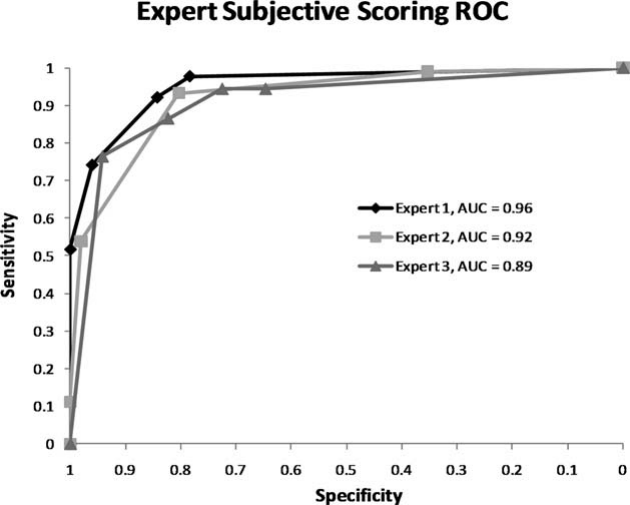
Subjective image analysis receiver operator characteristic (ROC) curves. Expert 1 is a head and neck clinical pathologist (M.D.W), and experts 2 and 3 are head and neck surgeons (A.M.G and V.S.).

Figure 2 shows the ROC curve for each of the 3 observers. A similar ROC curve was obtained for each observer. The average area under the ROC curve was 0.92.

### Quantitative Image Feature Calculation

**FIGURE 3:**
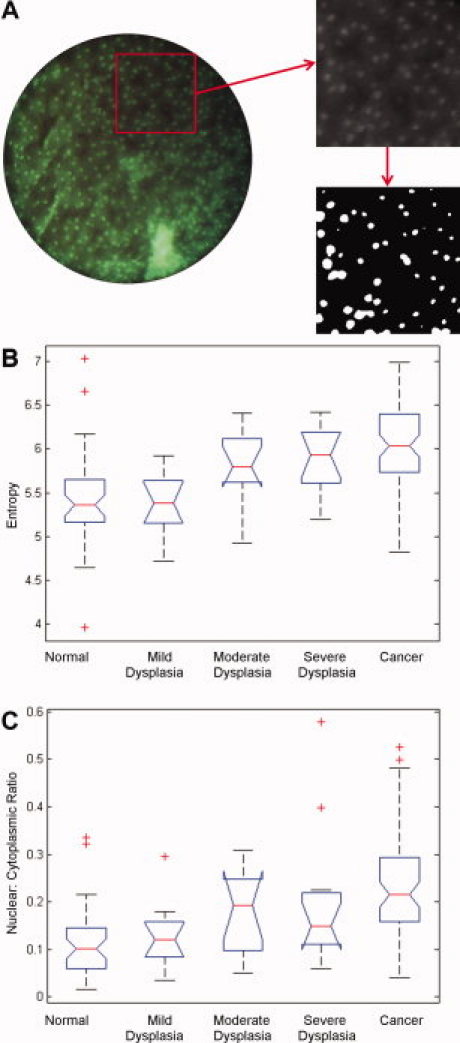
**(A)** Illustration of high-resolution microendoscope (HRME) region of interest (ROI) selection; note region containing visible cell nuclei selected by the observer. An example of semi-quantitative nuclear segmentation shown. Box and whiskers plots of **(B)** image entropy and **(C)** nuclear to cytoplasmic ratio by diagnostic category. The central line in each box represents the mean, whereas the top and bottom edges represent the 25th and 75th percentile. The notches in the boxes represent a 95% confidence interval for the mean.

**FIGURE 4:**
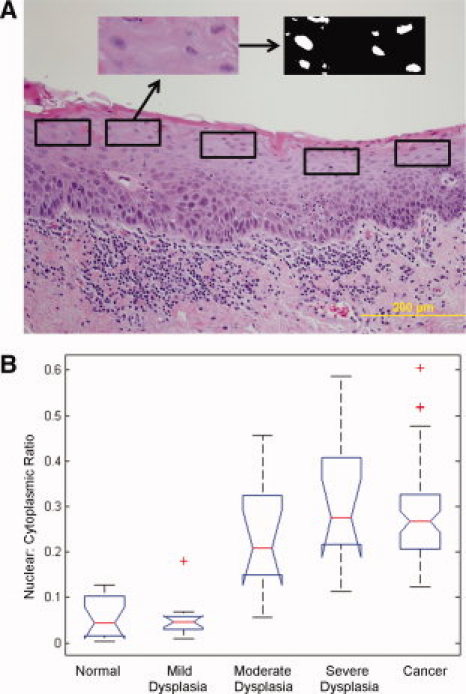
**(A)** Illustration of histology region of interest (ROI) selection. Each box represents an approximately 100 microns × 20 microns ROI. An example of semi-quantitative nuclear segmentation is shown. **(B)** Box and whiskers plot of nuclear to cytoplasmic ratio by diagnostic category.

A representative image showing a rectangular ROI selected from an HRME image from a site with a pathologic diagnosis of invasive carcinoma is shown in Figure 3A; the ROI was selected from a region of the image with easily recognized cell nuclei. Figure 3B and Figure 4C show box plots representing the calculated entropy values and nuclear-to-cytoplasmic ratio values for each ROI, averaged for each of the 5 histopathologic categories. On average, the entropy and nuclear-to-cytoplasmic ratio are higher for sites with moderate dysplasia, severe dysplasia, and cancer than for normal sites or sites with mild dysplasia.

The histopathology ROI review yielded 127 unique sites that closely matched the distribution of diagnoses imaged by the HRME (16 normal squamous, 11 mild dysplasia, 11 moderate dysplasia, 12 severe dysplasia, and 67 squamous cell carcinoma). A summary of the ROI selection process is shown in Figure 4A. A box plot summarizing the calculated nuclear-to-cytoplasmic ratio for each ROI is shown in Figure 4B; note the good agreement with the HRME results (Figure 3C), and the general trend of increasing nuclear-to-cytoplasmic ratio in moderate and severe dysplasia and squamous cell carcinoma.

**FIGURE 5:**
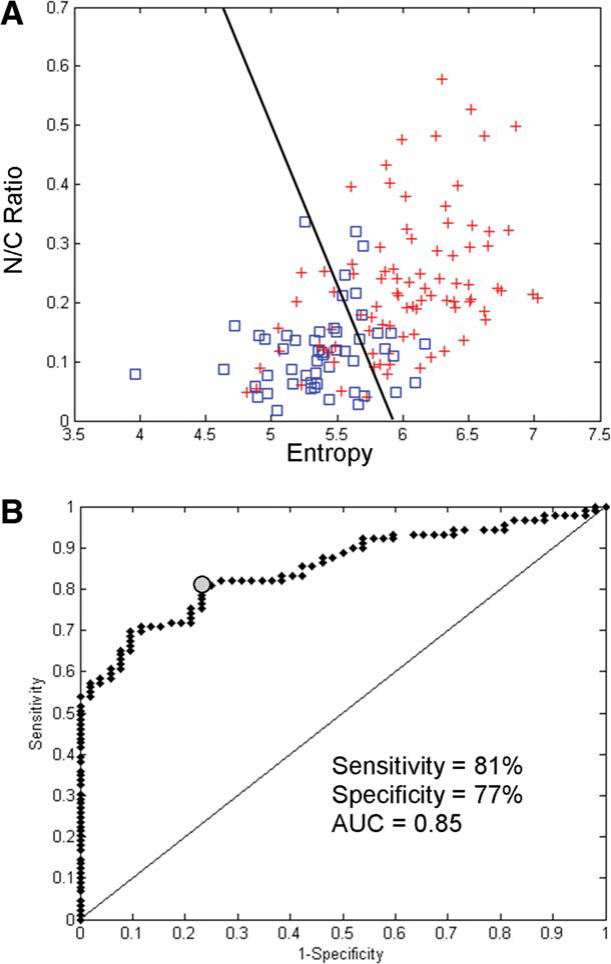
**(A)** Scatter plot of nuclear to cytoplasmic ratio versus entropy for all sites. Non-neoplastic sites are plotted as blue squares, neoplastic sites are plotted as red crosses. **(B)** Receiver operating characteristic (ROC) curve of the algorithm indicated by the discriminant line in **(A)**. The Q-point is shown, corresponding to a sensitivity of 81% and a specificity of 77%. [Color figure can be viewed in the online issue, which is available at wileyonlinelibrary.com.].

Figure 5A shows a scatter plot, indicating the entropy and nuclear to cytoplasmic ratio (as calculated from the HRME data) for each site. Histologically non-neoplastic measurements are shown as squares, whereas neoplastic sites are shown as crosses; the discrimination line separating the 2 classes is shown. Figure 5B shows the ROC curve for the linear discriminant classifier based on these 2 features. The area under the curve is 0.85 and the sensitivity and specificity at the Q-point are 81% and 77%, respectively.

## DISCUSSION

This study demonstrates the use of a robust, high-resolution microendoscope to image cellular and architectural features of oral neoplasia. Both subjective visual interpretation of images and objective analysis of digital images can be used to differentiate non-neoplastic and neoplastic oral mucosa. This suggests that HRME may have clinical applications for noninvasive assessment of oral lesions and at-risk mucosa.

Although these results are encouraging, there are a number of limitations associated with HRME imaging. First, the study presented in this manuscript was performed exclusively in an ex vivo manner. Future in vivo work is necessary to evaluate its clinical potential. Although no comprehensive long-term studies of the safety of proflavine as an imaging contrast agent have been published, this compound has a long history of safe clinical use in humans,^26^,^27^,^29^,^31^ and it has been used in a number of in vivo gastroenterology imaging studies.^27^,^39^

The surface imaging strategy used by this device cannot image submucosal tumors. In addition, significant hyperkeratosis or superficial necrotic debris may also preclude visualization of the cell layers that lie beneath. Hyperkeratosis can be seen as a layer of keratin on the superficial surface of the epithelium. This effect has been noted in other imaging studies using contact endoscopy and optical coherence tomography in a number of anatomic locations within the oral cavity, including the larynx and vocal cords.^40^–^43^ Because the HRME device requires that the distal tip of the image-guide fiber bundle be in direct contact with tissue, any material between the glass and tissue will obscure nuclear detail. In addition, the keratin layer seems to absorb some of the proflavine dye, which further obscures the nuclei below by introducing an undesired fluorescent signal. These technical limitations can potentially be overcome by 1 of 2 strategies: optical sectioning or mechanical penetration into deeper tissue. Optical sectioning, as used in confocal microscopy, enables an optical device to collect high-quality images from several hundred microns below the tissue surface.^17^,^44^,^45^ Alternatively, the fiber-optic bundle used in the HRME, which is less than 1 mm in diameter, can be inserted through the lumen of a 16-gauge hypodermic needle. In this fashion, the tip of the image guide can be directly inserted into deeper tissue, avoiding any keratin or debris layer at the surface. Such a technique has been previously demonstrated in an in vivo imaging study of a subcutaneous oral squamous carcinoma xenograft tumor.^22^ In practice, however, the examiner could simply choose to image a site adjacent to the area of hyperkeratosis, given that intraepithelial neoplasia usually extends at least several millimeters beyond clinically evident clinical lesions.^46^

Another limitation of the HRME is that only a small field of view can be sampled at a time. By combining this technology with either standard white-light examination or a wide-field imaging device, to quickly inspect the entire oral mucosa, the potential exists to increase the sensitivity and specificity obtained with either imaging method alone.

The proliferation of blood vessels in tissue stroma is seen in certain oral mucosa pathology, such as speckled leukoplakia and erythroplakia, and is associated with neoangiogenesis and tumor development.^47^ Detection of these changes would be helpful in improving detection of dysplasia. However, the HRME device has a depth of focus limited to the upper 20 microns of the tissue epithelium. Although this physical limitation enables high contrast visualization of nuclear architecture in the upper epithelium, it prevents the HRME from detecting subepithelial changes that are commonly seen in oral pathology, including angiogenesis, perineural invasion, and lymphatic invasion.

In conclusion, the HRME is a noninvasive, high-resolution imaging system well suited as a complement to other wide-field imaging techniques for detection and diagnosis of oral neoplasia. This low-cost device, in conjunction with an objective image classification algorithm, demonstrates the benefits of in situ high-resolution imaging of oral mucosa, and the potential to distribute this technology to a broad patient population.
